# Trust-building in temporary public health partnerships: a qualitative study of the partnership formation process of a Covid-19 test, trace and protect service

**DOI:** 10.1186/s12913-024-10930-3

**Published:** 2024-04-13

**Authors:** Eva Krczal, Doris A. Behrens

**Affiliations:** 1 Department for Economy and Health, University of Continuing Education Krems, Krems, Austria; 2 Employee Wellbeing Service, Aneurin Bevan University Health Board, Caerleon, UK; 3 School of Mathematics, Cardiff University, Cardiff, UK

**Keywords:** Collaboration, Partnership, Public health, Trust, Culture, Health care sector

## Abstract

**Background:**

Public health initiatives require coordinated efforts from healthcare, social services and other service providers. Organisational theory tells us that trust is essential for reaching collaborative effectiveness. This paper explores the drivers for initiating and sustaining trust in a temporary public health partnership, in response to a sudden health threat.

**Methods:**

This qualitative study analysed the formation process of a multisector partnership for a Covid-19 contact tracing service. Data was collected through 12 interviews, two focus groups, one feedback workshop, and an online survey with workforce members from all seven partner organisations. Purposive maximum variation sampling was used to capture the reflections and experiences of workforce members from all seven partner organisations. A deductive code scheme was used to identify drivers for building and sustaining trust in inter-organisational collaboration.

**Results:**

Relational mechanisms emanating from the commitment to the common aim, shared norms and values, and partnership structures affected trust-building. Shared values and the commitment to the common aim appeared to channel partners’ behaviour when interacting, resulting in being perceived as a fair, reliable and supportive partner. Shared values were congruent with the design of the partnership in terms of governance structure and communication lines reflecting flat hierarchies and shared decision-making power. Tensions between partner organisations arose when shared values were infringed.

**Conclusions:**

When managing trust in a collaboration, partners should consider structural components like governance structure, organisational hierarchy, and communication channels to ensure equal power distribution. Job rotation, recruitment of candidates with the desired personality traits and attitudes, as well as training and development, encourage inter-organisational networking among employees, which is essential for building and strengthening relationships with partner organisations. Partners should also be aware of managing relational dynamics, channelling behaviours through shared values, objectives and priorities and fostering mutual support and equality among partner organisations.

**Supplementary Information:**

The online version contains supplementary material available at 10.1186/s12913-024-10930-3.

## Background

Public health initiatives aim to promote health, prevent disease, and extend life through coordinated efforts by society, organisations, communities, and individuals [1]. This involves healthcare, social services, education, environmental protection, and non-governmental and voluntary organisations. To achieve effective coordination across the resulting organisational diversity, partnerships and alliances are established among (primarily non-profit) organisations within and outside the healthcare industry [2, 3]. However, establishing and maintaining collaboration requires continuous managed efforts [4] as organisations rarely share identical bureaucratic norms, rules, processes and structures [5]. A growing body of research has been conducted to comprehend the elements and processes that promote or hinder successful collaboration between healthcare organisations [6–8]. Empirical research has been presented predominantly from networks and collaborations that were established with a long-term perspective. There is scarce evidence of temporary cooperations that were formed to react quickly to an emergency situation. The recent Covid-19 pandemic exemplifies such a scenario, wherein public health providers had to quickly form cross-sector partnerships to contain the spread of the virus. This paper analyses a case where public health providers formed collaborative relations at an exceptionally fast pace to respond to a sudden health demand.

Specifically, this study explores trust building in a temporary partnership. In a collaborative setting, perceptions of trust as well as implicit norms of reciprocity and mutual support typically play a central role in guiding partner organisations’ interactions [9]. Trust has been identified as a critical determinant of collaborative success [10–13], where “success” refers to effectively reaching health and health system goals [14]. Once trust is established among collaborators, time and effort can be freed to achieve collaborative synergies, such as pooling resources and knowledge, sharing best practices, and developing innovative healthcare solutions [15].

Organisational theory addresses trust-building in collaborations as a reciprocal relationship between trust and risk tolerance [16–18]. Vangen and Huxham propose trust building as a reinforcing loop [19]. From their model’s perspective, organisations rely on their expectations about partner behaviour and collaborative outcomes to decide whether to (further) engage in a partnership. Vangen and Huxham [19] suggest that trust increases incrementally through partners’ interactions. These interactions shape expectations about cooperative or opportunistic behaviours [17], where expectations solidify when met and subsequently affect actions (which in turn affect expectations, and so on). In the case of incrementally solidifying the expectation of cooperative behaviour, trust is being built between partners [16]. Likewise, trust is reinforced whenever expected collaborative outcomes are observed [19]. Therefore, it has been recommended that organisations set modest goals at a collaboration’s onset to achieve “first gains” swiftly. These quick wins then boost confidence in the collaboration and its benefits [20]. When actors individually and collectively believe that the collaboration will be beneficial, they are willing to dedicate time and effort to engage in collaborative behaviours [21].

Vangen and Huxham further introduce the trust-building loop as a conceptual and pragmatic tool [19]. They propose five management themes dedicated to initiating and sustaining trust-building. Those management themes are summarized in Table 1.

**Table 1:** Trust management in inter-organisational collaboration

Dimensions	Management themes
**Initiating the trust-building loop**	**Forming expectations** Involves gaining clarity on central members, their roles & status and reaching an agreement on the collaboration’s aims and purpose.
	**Managing risk** Involves understanding the aims and agendas of partners, ensuring the ability to enact the collaborative agenda and that future collaborative gains are shared. Ensuring that collaborative gains can be obtained, for example by aiming for realistic (initially modest) but successful outcomes.
**Sustaining the trust-building loop**	**Managing dynamics** Involves dealing with changes in purpose, membership, the structure of the collaboration, and external factors (government policies, funding).
	**Managing power imbalances** Involves ensuring shared power, understanding changes in the balance of power, and dealing with perceptions of power inequality.
	**Nurturing collaborative relationships** Involves continuous management efforts to sustain trust: paying attention to the management of communication, power imbalances, recognition of partner’s contribution, common ownership, commitment to the collaboration, re-negotiation of aims & agendas

Research on inter-organisational healthcare collaborations suggests that trusting attitudes need time to develop pointing to the incremental element of trust-building [13]. Our case study provides the opportunity to test the trust-building loop for a temporary collaboration in an emergency situation. Building on the conceptual framework from Vangen and Huxham (Table 1) we explored trust-building in a British contract tracing service formed in May 2020 as a multisector partnership to respond to Covid-19 challenges. Partner organisations rapidly formed collaborative structures and established inter-organisational teams facing time pressure, and uncertainty and pursuing ambitious goals from the outsets. Through the lens of the “trust-building loop” [19], we examined the critical drivers for initiating and sustaining trust in a temporary collaboration, in response to a sudden health threat.

## Methods

### Study design

The present research was part of a more extensive, qualitative exploratory case study to obtain in-depth information on the management and leadership issues involved in the partnership formation process [22]. We used a qualitative approach to understand partner organisations’ views on enablers and barriers to reaching collaborative effectiveness [23]. This paper focuses on one facet when striving for collaborative effectiveness: the trust-building in a public health partnership.

### Research site and context

This study examined the collaboration between Aneurin Bevan University Health Board (ABUHB), Public Health Wales (PHW) and five county borough councils (Blaenau Gwent, Caerphilly, Monmouthshire, Newport and Torfaen). The overall objective of the collaboration was to rapidly create a test, trace and protect service in South Wales, UK, to break the chains of SARS-CoV-2 transmission within the Gwent population (roughly 600,000 people). The partnership between the local health board and Gwent’s local authorities sought to establish regional and local contact tracing structures, manage pooled resources, and coordinate the joint pandemic policy (following Welsh-Government guidelines). In peak times, the staff comprised more than 400 employees recruited (or temporarily deployed) from public service providers and the private sector. For a detailed description of the set-up, evolution and culture of the Gwent Test Trace Protect Service (GTTPS), see Behrens et al. [22].

### Participant selection and recruitment

The case study analysis rests on a cooperation agreement between the University for Continuing Education Krems and the Aneurin Bevan University Health Board. The Aneurin Bevan University Health Board (ABUHB)’s Research and Development Department has approved all study activities under R&D reference number SE/1338/21. The selection of study participants (across all partner organisations, accounting for gender parity) followed a maximum variation sampling approach [24]. I.e., interviewees and workshop members were purposefully sampled from leading positions of member organisations. In contrast, focus group participants were sampled from diverse professions and job roles to gather information on partnership members’ (different) perspectives and interpretations at the lower end of organisational hierarchies [25]. Interviewees, focus group participants and workshop members were invited via email to join an MS-Teams meeting, receiving a short explanation of the purpose and a suggested date. All invited persons agreed to participate. Verbal informed consent was obtained prior to starting the interviews or focus groups.

Additionally, the service’s project management office at ABUHB invited 570 current and former GTTPS team members to participate in an online survey. Respondents were mostly current employees. The response rate was around 33%. For more information on the study participants, see Table 2.

**Table 2:** Study participants by partner organisation

Partner organisation	Interviewees	Focus group members	Feedback Workshop	Survey participants^a^
ABUHB	3	7	3	16
Public Health Wales		1		4
Gwent Local Authorities^b^	9	5	5	50
Total	12	13	8	70

*ABUHB *Aneurin Bevan University Health Board

^a^For this study, only data from one (of two) open survey questions was analysed (which was answered by *n* = 70 GTTPS team members)

^b^The Gwent Local Authorities also hosted the Coordinating Unit with the “Head of Service”

### Data collection

Data were collected over five months from Oct. 2021 – Feb. 2022 through 12 semi-structured interviews, two focus group discussions with 13 participants, one feedback workshop with eight participants, an online survey, and documentary evidence.

EK and DB conducted semi-structured interviews and focus groups. The interview guide focused on participants’ reflections on their working experience within the GTTPS and covered three main themes: organisation, communication and leadership. Study participants were encouraged to reflect on factors that facilitated/hampered collaboration, benefits, drawbacks, and how critical issues were addressed. This exploratory and non-directive questioning method was used in previous research on the dynamics of trust in collaborations [26, 27]. The researchers conducted all interviews and focus groups via Microsoft Teams. Individual interviews lasted 30–45 min; focus groups lasted around 90 min. A final 90-minute feedback workshop on preliminary findings was used to consolidate the results and collect further data. DB conducted the workshop via Microsoft Teams and recorded information as unstructured notes.

The questionnaire comprised 65 closed and two open questions and was conducted using Microsoft Forms, with responses from a third of the service’s current and past workforce ($n=188$). The 70 non-empty answers (37%, $\stackrel{-}{n}=70$) to the open question, “Is there anything else you wish to share about how GTTPS has developed?” were used for this study. EK screened this single item for themes relating to trust-building. The full survey results can be found in [22].

Documents were gathered from people involved in the planning and implementation of the GTTPS. Documentary evidence covered the governance framework, internal reports, job interview questions, and training programs. The documentary evidence complemented (and verified) study participants’ narrations of their experiences and perceptions of the partnership.

### Data analysis

Interviews and focus-group discussions were audio-recorded and transcribed externally. Transcription was done verbatim, with annotations following Dresing and Pehl’s recommendations [28]. Content analysis was applied to produce a structured and comprehensive data set reflecting study participants’ perceptions and opinions on the critical drivers for initiating and sustaining trust. Specifically, we conducted a qualitative content analysis by extraction [29]. This method follows the common procedure of content analysis involving categorising, extracting and consolidating information to address the research question [30]. Typically for the content analysis by extraction, the extracted data also included information about how categories were connected and the study participants’ reflections on the causes and effects of a specific issue [31]. This procedure facilitated an exploration of the interplay between the categories providing us with a comprehensive insight into the drivers for initiating and sustaining trusting partnership relations [32]. Five main categories formed the basis for structuring the data. These main categories were defined deductively based on the management themes proposed by Vangen and Huxham on how to initiate and sustain the trust-building loop (see Table 1).

The first author assigned text segments from the interviews, focus groups and the open question from the survey to the main categories. Next, the first author inductively created sub-categories to develop a more refined data set. Sub-categories were defined according to central themes that emerged within each main category. Next, qualitative data was coded in a second round along the sub-categories. During the coding process, preliminary findings and interpretations were compared and blended with existing theory, moving toward higher levels of theoretical abstraction [33]. Coding was refined in an iterative process that involved the introduction of new sub-codes or the re-evaluation and re-definition of existing sub-codes. It was decided to delete the main category “Managing dynamics”, sub-codes were integrated into the category “Managing power-imbalances”. The modification in the coding scheme was more appropriate for this case. As a temporary partnership, the service was disbanded when the purpose and funding faded. A sample data extraction sheet used for this analysis can be found in Additional Table 1 (Additional file 1).

Categories and their relations were discussed with other members of the research team. In a final step, the researchers visualised and discussed ‘emerging’ drivers for initiating and sustaining trust. Recognising their role in reconstructing and interpreting qualitative data, the researchers applied the following techniques recommended by the literature to enhance the validity of findings [29, 30, 34]: (1) The researchers collected data from multiple sources (interviews, focus groups, feedback workshops, survey participants and documentary evidence). (2) DB spent prolonged time (21 months) in the field conducting interviews, obtaining feedback from study participants and observing interactions between partnership members. (3) A journal continuously documented the research process (including developing the coding system and analytic reflections). (4) Peer debriefing with other researchers was used to discuss emerging findings and patterns.

## Results

The content analysis yielded six key themes and 15 subthemes epitomising enablers for trust-building (see Table 3). A summary of the trust-building processes emanating from the content analysis can be found in Additional Table 2 (Additional file 2).

**Table 3:** Trust-building elements resulting from the content analysis by extraction [31]

Dimensions	Themes and Subthemes
**Initiating the trust-building loop**	**Forming expectations** Previous experiences of collaboration Need for collaboration Commitment to a common aim
	**Managing risk** Collaborative set-up of service
**Sustaining the trust-building loop**	**Managing power imbalances** Governance structure Shared decision-making Dealing with power imbalances Dealing with tension and failure
	**Nurturing the collaborative relationships** Network communication Approachable, supportive workforce Situational awareness Recognition and respect Gaining underpinnings for more ambitious collaboration

### Initiating the trust-building loop

#### Forming expectations

The outcome of partnership decisions was heavily influenced by experiences gathered in former collaborations, situational circumstances, and a shared dedication to a common aim. The collaborators emphasised that established personal relationships and prior connections between the organisations facilitated the partnership formation process. One focus group participant explained:

*“There were some existing good relationships with the [other] organisations that we were sort of able to build on to get the agreement for establishing the service.” [Participant Focus Group 2]*.

Study participants noted that partner organisations collectively aimed to safeguard their communities and maintain the smooth functioning of services. Facing the same challenge (like making decisions under uncertainty while coping with limited resources and know-how) further united the partners. For example, one participant stated:

*“I think that position of everybody not knowing what’s going to happen was quite unifying. […] It wasn’t that somebody knew what was going to happen, and other people didn’t. We were all in the same boat.”* *[Interviewee 10]*.

The collective effort and commitment towards the collaboration were highly evident and appreciated by the participants, who perceived a strong sense of dedication towards the common goals. One participant specifically emphasised *“the willingness of [all] partner organisations to participate in the planning and the implementation and the further improvement of that service.”* *[Interviewee 3]*.

One of the focus group participants expressed how working together with a common goal created a sense of camaraderie and connection. The following quote exemplifies the bonding experience that resulted from shared commitment and goal orientation:

*“There are six teams working together across Gwent, and at one point, it was over 500 employees that all work together for the common aim. And that’s what brings us together, and in our daily chats, we always put #TeamGwent – one team, and that’s what we are. We are one team working together for a common goal.” [Participant Focus Group 1]*.

One feedback workshop participant remarked that partner organisations aligned objectives and prioritised goals in the same order to safeguard their communities. The feeling that everyone was working in the same direction was referred to as the *“glue”* between partner organisations *[Interviewee 4]* and created a *“big team feeling”* *[Participants Focus Group 1 & 2, Interviewee 6]*.

The backing of Human Resource (HR) strategies and leadership reinforced the dedication to the common aim. Internal documents on the leadership development program revealed that NHS leaders were strongly advised to instil a shared sense of purpose and promote connections within the service. According to the study participants, leaders were role models demonstrating commitment and collaboration towards shared goals. For example, one participant used terms like *“working extremely hard”* and *“genuinely”* trying to do *“the right thing for Gwent”* to describe leaders’ commitment to the common aim *[Interviewee 8]*. Besides, the inaugural team of spring 2020 brought together people from various organisations who mainly volunteered to work for the service in the making. Thus, Human Resource strategies have successfully attracted and pre-selected people highly committed to the service and its purpose.

#### Managing risk

The rapid and structured set-up of the service was associated with partnership effectiveness. One participant reflecting on the establishment of the contact tracing service stated that it was *“incredible [to see] the way in which organisations came together to set up the service”* and highlighted *“the amazing progress that was made, very, very rapidly when the service was first established.”* *[Interviewee 3].*

This “first gain” emerged from data analysis to have strengthened faith in the collaborative functioning of the service. Study participants considered collaboration, the ability to work together as a team, as a crucial success factor of the service: *“[…] the key success for me is just their ability to pull together and get something together.”* *[Interviewee 6]*.

Another participant added: *“[…] because that’s the learning from COVID: the more collaboration, the better the job is done. Whatever the job is.” [Interviewee 2]*.

### Sustaining the trust-building loop

#### Managing power imbalances

Insights gained from data analysis showed that partner organisations strived for equal power distribution. The individuals who took part in the study highlighted the absence of hierarchical thinking and appreciated equal representation, decentralised structures and local control. The GTTPS governance framework^1^ promoted equality among the partners, and our analysis showed that the power distribution within the service followed suit. The Regional Oversight Group played a significant role in strategic planning and coordinating activities. It was referred to as a *“collective”* or *“shared decision-making forum”* with participation from all partners, who were encouraged to express their views *[Interviewee 8, Interviewee 2]*. Further, study participants explained that the Coordination Unit, responsible for managing contact tracing across the five councils, moved from the health board to a local council to avoid public service partners *“feeling like they were being dictated or controlled by the health board”* *[Interviewee 1]*. Also, the Head of Service, who led the unit, was recruited from within the councils—with the Coordinating Unit offering support instead of enforcing authority or control.

At the formation stage, some participants noticed tensions between the partner organisations concerning the service’s design and structure. At the same time, these participants reported that the partner organisations overcame these tensions by collectively developing a road map to working together. One participant outlined:

*“[…] Consensus decision-making is vitally important, and that is what happens at our governance groups, the Regional Oversight Group, the operations groups. […] There are people in leadership positions reporting to other people in leadership positions in different organisations. That simply does not happen in very many places, does it?” [Interviewee 2]*.

According to study participants, decision-making resulted from frank discussions where contributions from all members were recognised. One participant stated:

*“[…] all team members are allowed to put their contributions in. I think that it is really important that any team member, irrespective of where they are in the hierarchy, is allowed to have a say because that is the only way a set-up like this can work.” [Interviewee 9]*.

Data analysis revealed some inequalities regarding central control versus locally-based decision-making. One participant outlined:

*“I expressed these views, […] until I was told to shut up, which is absolutely fine. I was heard. My views, my concerns were heard again. This was again […] the governance issue. It is the central control versus local, and you know we were, we were told … it was made clear that we will be part of the group, and we were expected to abide by it, which we largely did.” [Interviewee 8]*.

Increasing bureaucracy led to tensions between partner organisations, particularly in the mature phase of the partnership. Other examples of tensions indicated by study participants derived from a lack of acceptance of particular professions or when one partner claimed not to receive sufficient information, explanation or support from another partner. When the service was under pressure, one interviewee noted a tendency to prioritise the needs of the own sending organisation, which challenged collaboration with this partner.

Partner organisations tried to overcome such tensions through frank discussions, explaining the reasons for decisions and showing interest in others’ views and opinions. Understanding people’s behaviours and attitudes was considered essential to get to the bottom of the tension’s cause. One participant explained:

*“[…] it could be quite tough […] the atmosphere could be tense, at times. You know, sometimes asks were made; I think I would say they were not reasonable because they had not been thought through. So, I think the challenge was trying to get to the bottom of that. Trying to work out where people were coming from and to use that as we have a constructive dialogue*.” *[Interviewee 9]*.

Trust was considered a foundation for this open and problem-focused discussion climate:

*“We don’t always agree, we don’t always get on, but there is trust.” [Interviewee 8]*.

Participants described a partnership culture where failures were not stigmatised. One participant expressed: *“We need to accept that things can go wrong. That no system is ever perfect. […] Understanding the pressures people are under is another lesson to be learned.”* *[Interviewee 9]*.

Indeed, failures were considered an opportunity to learn and adapt. The resulting organisational climate made staff feel confident and trusted. Study participants outlined throughout the interviews that they were not afraid to admit mistakes or discuss challenging issues. One study participant explained:

*“But occasionally they would get it wrong, and I think it is having the confidence, […] feeling confident and trusted […] that it wasn’t the end of the world*.” *[Interviewee 8]*.

#### Nurturing collaborative relationships

Our analysis confirmed that the study participants placed great importance on effective communication for building trustworthy relationships. They highlighted the significance of frequent formal and informal meetings, job rotations, and non-hierarchical service structures in facilitating seamless communication within and between organisations. A participant emphasised the relevance of spending time together to nurture empathy and trusting relationships:

*“[…] We know each other reasonably well, and we’ve established that through our regular team meetings and our morning catch-up. […] a bit like a well-worn suitcase where we are comfortable with each other. We don’t always agree, we don’t always get on but – but – but there’s a trust there, […], and it has developed through time as we got to know each other better. Having spent quite a lot of time in each other’s pockets*.” *[Interviewee 8]*.

The study participants stated that the workforce exuded a welcoming and supportive environment throughout the partnership network, spanning from top executives to team members across all partner organisations. Emanating from the service’s governance framework, which identified *“mutual aid”* as a fundamental principle, study participants highlighted that they felt surrounded by a support network which induced positive working experiences and high-quality relationships between service members. One survey participant outlined: *“I feel no barriers between each team and would happily seek support and advice from any partner”* [Survey Participant 89]. Similarly, an interviewee mentioned that *“there is certainly that support network across Gwent TTPS that we can call upon. And we built up some fast, fantastic […] working relationships” [Interviewee 6].*

This collaborative and supportive culture was promoted by the innate characteristics and values of leaders and employees and a shared commitment to a common goal. Partners’ willingness to widely share information, advice, expertise and support when managing their workload was paramount. One participant remarked:

*“I would have no hesitation to phone up somebody of the leadership team from the health board if I needed to know something, whereas that’s not traditionally what I have witnessed before. I think, you know, it is strong leadership, and that’s down again to personalities and down to the focus and commitment and passion. But they are also very, very approachable leaders.” [Interviewee 11]*.

Meetings were described as two-way communication focusing on solving problems, connecting service members, creating understanding for decisions and actions and creating “*situational awareness*” by keeping everyone informed and involved *[Interviewee 3]*.

The free exchange of information and advice supported mutual recognition and respect. In sharing knowledge and expertise and discussing problems, partners demonstrated their professional competence and willingness to help each other. One survey participant outlined:

*“The TTP service was created through passionate people with a variety of skills and experience. […]. People worked at pace and trusted each other to take on activities within their expertise and then develop knowledge and skills for each other. The recognition of people’s strengths really did help get the service implemented.” [Survey participant 89]*.

Leaders were described as (acting like) role models by accepting each other’s professional roles but also claimed this attitude from team members. The following quote reflects leaders’ attitudes:

*“And often I would have to intervene and say yes, you are part of the team, but there are other regional players in this, and it’s important that you accept that […] and acknowledge the role they have been playing.” [Interviewee 9]*.

Further, common gains strengthened partner organisations’ confidence in the collaboration and its benefits. The partnership’s high performance during peak infection times confirmed participants’ perceptions of collaboration effectiveness. One interviewee outlined:

“*[…] our performance has been incredibly high reaching, you know 90 odd percent of people within 24 hours”* and added, *“to be able to do what we’ve done in such a short space of time whilst keeping a level of governance, I find truly outstanding.” [Interviewee 2]*.

Besides, study participants experienced knowledge gains and personal learning opportunities attributed to collaborative synergies. A focus group member highlighted the following:

*“We have seen some amazing innovation and collaboration across departments, and I think those communication skills and adaptability skills as something that can be really built upon and used in future programs of work.” [Participant Focus Group 1]*.

## Discussion

This study explored drivers for building and sustaining trust within a public health partnership of non-profit organisations. The study used a theoretical framework (19) as a starting point for analysing factors contributing to the partnership’s trust-building process.

Findings suggest that the foundations for trust for the present collaboration were established before and during the formation stage of the partnership. Previous positive experiences in collaboration and the perceived need for a collective approach to strive for the shared aim evoked partners’ commitment to the partnership. Amid the unpredictable context of the pandemic, partner organisations demonstrated a willingness to collaborate, share knowledge and consider others’ perspectives and opinions in making joint decisions to decide on the best way of action. Partner organisations entered negotiations about forming a partnership by displaying cooperative behaviours. Initial tensions that arose from discussions concerning the design and structure of the service were solved by consensus, i.e., no partner appeared to dominate the decision-making process. These interactions between partners appeared to generate initial confidence in partners’ behaviours and expected outcomes of the collaboration.

As proposed by the trust-building loop, we observed that first gains increased faith in the collaboration and partners’ behaviour. The positive effect of first gains on trust in collaboration has been demonstrated in previous research [35]. Participants of this study considered first gains proof that partner organisations could collaborate effectively and achieve the common aim of the service. More ambiguous gains, such as high performance during peak infection times or organisational learning, reinforced faith in collaboration and partners’ behaviour.

Our findings additionally highlight the role of shared values in sustaining the trust-building loop. These shared values appeared to be continuously nurturing the collaborative relationships between partner organisations. The connection between shared values, organisational culture and trust has been addressed in previous research. Similar norms, values, beliefs, and collaborative organisational cultures are expected to reduce conflict and increase confidence [36–38].

Our results suggest that the first gains not only increased faith in the collaboration and collaborative behaviour of partners but also supported the development of a distinct partnership culture. When developing partnership structures and relations, partner organisations were guided by certain behavioural norms and values that have proved successful during the formation stage of the partnership. According to Schein, the organisational culture evolves from decisions, actions and behaviours that proved successful when solving problems by working together or adapting to external circumstances [39]. Our findings align with this by indicating that the core values of the service represented critical success factors for service effectiveness, indicating that these shared values have induced the evolution of a partnership culture.

**Fig. 1:**
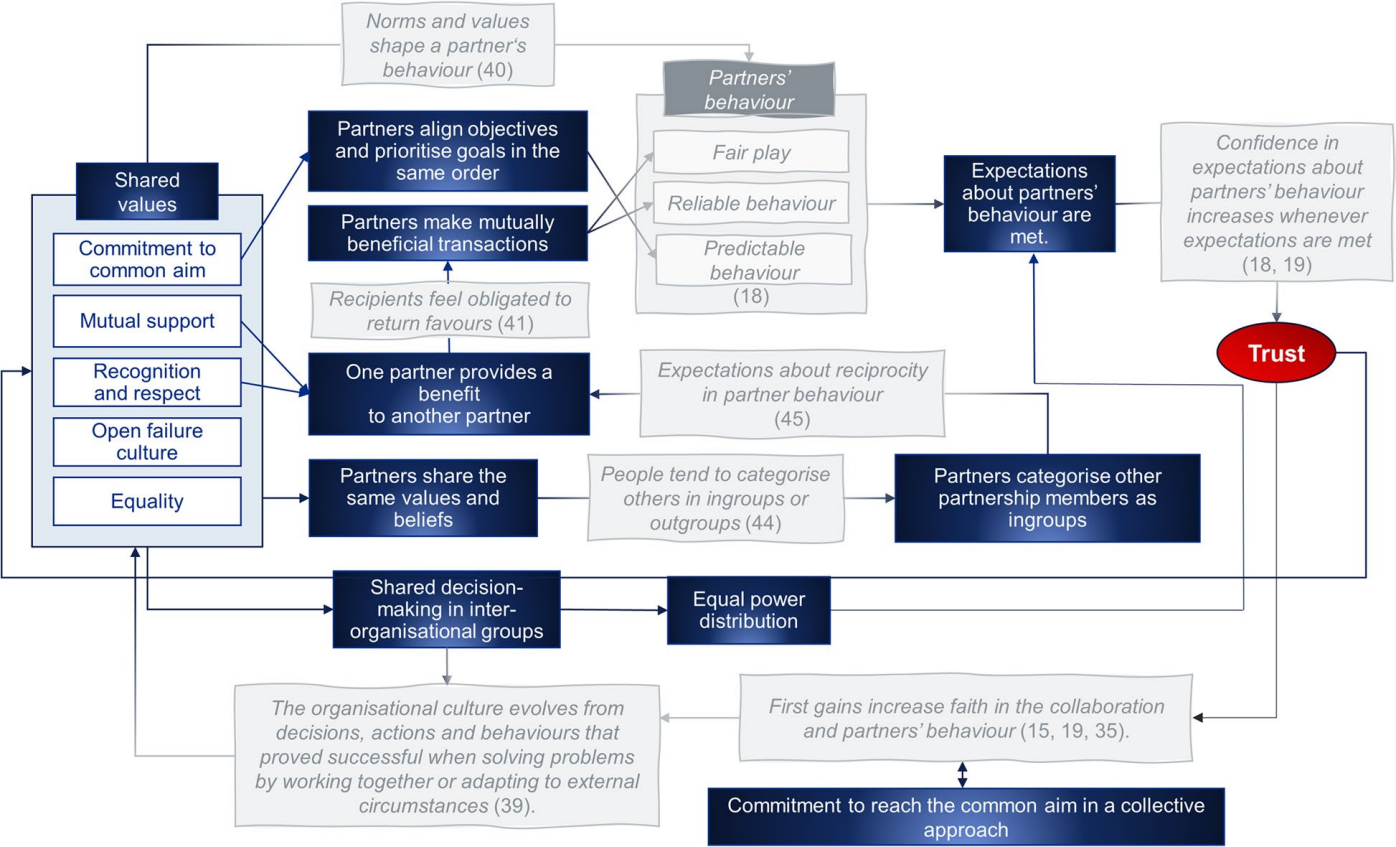
Trust-building processes induced by shared values observed in the study and blended with theory

The five features of the partnership culture observed in this study are (1) commitment to the common aim, (2) mutual support, (3) recognition and respect, (4) equality, and (5) open failure culture. Figure 1 illustrates the trust-building processes induced by the shared values that have evolved from data analysis blended with the organisational and behavioural theory to explain the mechanisms observed in our study. Boxes in grey and with odd lines represent the theoretical foundation for the process of trust-building. Boxes in blue and with flat lines represent mechanisms that were uncovered through analysis of the empirical data from this case study.

Collectively shared norms and values are suggested to shape members’ behaviour [40]. According to Zaheer et al. [18], trust is the expectation about a partner’s behaviour concerning three components: reliability, predictability and fairness (see Fig. 1). Developing a shared partnership culture thus increases the reliability and predictability of each partner organisation’s behaviour, producing higher levels of trust whenever expectations about partners’ behaviour are met [18]. Besides, the high commitment to the common aim encouraged partners in our study to align objectives and prioritise goals in the same order. Pursuing the same priorities and objectives might also channel behaviours, thus increasing the reliability and predictability of partners’ behaviour.

Next, participants stressed mutual support and open exchange of information and expertise between partner organisations, indicating reciprocity and mutual exchange of benefits in partners’ interactions. The results align with the Social Exchange Theory, which proposes that trust develops through mutually beneficial (trans)actions [41, 42]. This means that when one party provides a benefit to another party (e.g., professional expertise, social support, recognition), the recipient feels obligated to return the favour. Consequently, trust is built iteratively through a consistent exchange of benefits and broadening interactions between the parties [43]. Hence, the principle of reciprocity should increase confidence in expectations about partners’ behaviour and the outcomes of the collaboration.

Moreover, study participants noted that striving for the same aim and sharing the same values and beliefs evoked a “*big team*” feeling. According to Social Identity Theory, individuals tend to categorise themselves and others into subgroups based on sociocultural distinctions [44]. When one party perceives that the other party is similar to them in some way, such as sharing the same values or beliefs, they will be categorised as ingroup members. Shared ingroup membership is suggested to increase expectations of reciprocity which in turn reinforces trusting behaviours [45].

We also analysed the impact of the power structure on trust-building processes. Findings demonstrate that the principle of equality guided partner organisations’ interactions through consensus decision-making, respecting partners’ contributions and opinions, and sharing information and expertise. We could also observe a fit between organisational culture and structure in a way that the principle of equality guided partner organisations when designing governance structures and communication channels. First, the service and governance structures of the partnership were characterised by an absence of rigid hierarchies. Second, governance structures were organised in several inter-organisational groups, such as information-sharing and joint decision-making forums. In this regard, we can assume that the principle of equality and the partnership structure reinforced partners’ cooperative behaviours. Previous research has documented that the power structure is related to resource distribution in collaboration [46], thus affecting expected outcomes and the level of trust in a collaboration [47]. For example, in analysing cases of a healthcare alliance, Murray et al. [48] reported that expectations on using funds and providing expertise affected trust in a partnership. When expectations were not met, this led to a reduction of trust.

Besides, our results demonstrate that leaders played an important role in channelling behaviours directed towards the common aim and acted as role models for cooperative behaviour. Our study reiterates findings from existing research on collaboration effectiveness, stressing the importance of leadership for engaging, mobilising and involving all partners [49, 50].

Supporting previous research [47], our study results stress the importance of frequent formal and informal communication for building relationships and trust in collaboration. Findings indicate that organisational structures, and specific HR-Strategies such as job rotation and recruiting candidates with the desired personality traits and attitudes, encouraged network communication across partner organisations. Similar HR strategies, including training and development, have been addressed before to promote inter-organisational networking [51].

In the present study, we observed that the shared values strengthened partnership relations on the one hand. On the other hand, these shared values jeopardised the partnership if they were not observed. Study participants noted tensions between partner organisations when one partner perceived a violation of shared values by another partner. For example, when one partner provided only insufficient information to the other partner, the value of “*mutual support*” was not shared. The infringement of this value caused tensions between partners as expectations about partners’ behaviour (being supportive) and the outcomes of the collaboration (receiving information) were unmet.

### Strengths and limitations

This study was a qualitative case study to obtain an in-depth understanding of the drivers for building and sustaining trust from the perspective of service members. The explorative approach allowed us to identify and understand in detail the trust-building processes emerging from the experiences and reflections of participants. To the best of our knowledge, there is no research on how the underlying mechanisms of shared values and beliefs affect trust-building in a public health collaboration. Our results provide insights into this issue. However, some limitations have to be considered when interpreting results.

The purposeful sampling strategy might have induced a selection bias, when individuals highly committed to the partnership were nominated to participate in the interviews and focus groups. Further, the enthusiasm expressed by many study participants might have prompted a positivity bias, favouring positive associations with the partnership experience. Several situational factors appeared to influence the collaborative behaviour of partnership members. The unprecedented level of uncertainty, the perceived need to consolidate efforts, and the dynamic work context might have prioritised the need to collaborate. Further, study participants stressed that the funding was not a limiting factor in the partnership as there was a firm funding commitment from the Welsh Government for the service. This situation differs from cases analysed in previous research where funding commitment or the distribution of funds have been reported as substantial sources of conflict and may ultimately lead to the collaboration’s end [48, 49]. The uniqueness of contextual factors poses limitations on the generalisability of findings. On the other hand, unconventional research contexts or samples can offer novel insights and pose new impulses for research [52].

Further research on the impact of shared values on trust can contribute to a better understanding of collaboration functioning. For example, the features of the partnership culture identified in this study point to a psychologically safe working climate. The concept of psychological safety has been developed and studied in the context of team learning and team performance [53–55] and is also considered essential when forming temporary teams [56]. Analysing trust-building in the context of psychological safety would enrich findings on the interplay between shared values and trust.

Some of the partnership features described in this study might challenge traditional management principles of public health organisations, especially those with a high degree of formalism and hierarchical orientation. Our findings echo research on organisational learning, stressing that it needs flat hierarchies to foster innovation and adaptability [57]. Future research in this area can study organisational learning in a broader context by exploring potential ‘spillover’ effects from learning experiences deriving from inter-organisational collaborations with partner organisations.

## Conclusion

Guided by a theoretical framework, this study analysed the drivers for building and sustaining trust in a public health partnership. Results of this study suggest that relational mechanisms emanating from the commitment to the common aim and the shared norms and values, as well as partnership structures, affect trust-building. These factors appeared to channel partners’ behaviour when interacting, resulting in being perceived as a fair, reliable and supportive partner. In our study, shared values are congruent with the design of the partnership in terms of governance structure and communication lines reflecting low hierarchies and shared decision-making power.

When managing a collaboration, partners should pay attention to structural components to ensure equal power distribution in a partnership. For example, equal representation of all partner organisations and/or a rotating chair in strategic decision-making forums can ensure (the perception of) equality. Partner organisations are also advised to ensure smooth communication channels and encourage informal communication between partner organisations to support information exchange and mutual understanding of partners’ decisions and behaviours.

Further, partners should be aware of the role of leadership in encouraging commitment to the common aim and collaboration. The promotion of cooperative leadership styles can support trust-building processes in a partnership. For example, servant leadership has been found to promote trust, fairness and reliability in a relationship because of the enhanced empowering and supportive behaviours of the leader [58]. Recruitment and training strategies can be designed to support inter-organisational networking and collaboration among the workforce. Selecting people with traits like being supportive, committed, and open to other opinions could be a recruitment strategy supporting a culture of collaboration. Job rotation can be used as a personnel development measure to expand personal networking and increase mutual understanding.

### Supplementary Information

**Additional file 1. **Data Extraction for “Managing risk”, “Collaborative set-up of service”. Sample data extraction sheet for theme “Managing risk “Collaborative set-up of service”.

**Additional file 2. **Summary of trust-building processes emanating from content analysis. Summary of drivers for initiating and sustaining trusting partnership relations resulting from an exploration of the interplay between different variables extracted from data analysis.

## Data Availability

The datasets generated and/or analysed during the current study are not publicly available due to potential confidentiality concerns but are available from the corresponding author on reasonable request.

## Abbreviations

ABUHB: Aneurin Bevan University Health Board
e.g.: for example
GTTPS: Gwent Test Trace and Protect Service
HR: Human Resources
i.e.: that is
PHW: Public Health Wales
R&D: Research and Development

## Footnotes

1 The Contact Tracing Governance and Delivery Framework represents the collaboration agreement between partner organisations. It contains the purpose, collective aim and objectives of the service, the principles of the service, the organisational structure of the service, and the roles and responsibilities of partner organisations and groups. The latter formed the governance structure of the partnership: strategic planning and decision-making was executed in several groups with representatives from partner organisations.
