# Supplementary material for: Trust-building in temporary public health partnerships: a qualitative study of the partnership formation process of a Covid-19 test, trace and protect service
Source: BMC Health Serv Res. 2024 Apr 13;24:467. doi: 10.1186/s12913-024-10930-3 (PMC11015697; doi:10.1186/s12913-024-10930-3)
Supplement: Supplementary file 1 — Additional file 1. Data Extraction for “Managing risk”, “Collaborative set-up of service”. Sample data extraction sheet for theme “Managing risk “Collaborative set-up of service”. [file 12913_2024_10930_MOESM1_ESM.pdf]

Additional Table 1 Data Extraction for “Managing risk”, “Collaborative set-up of service”

Stage*	Cause	Theme	Effect	Source
1		The collaborative, supportive nature of the set-up of service is seen as a success	Team view incorporates all partners<br>(Big team feeling)<br>(Faith in collaboration)	I9: 3
1	Each partner contributed expertise<br>Strong project management	A rapid and structured way to set up a service	Successful governance structure<br>Highly effective	I11: 8
1	Commitment to partnership and common aim	High speed of response	(Feeling that ability to work together is the key success of service)	I11: 16
1	Commitment to partnership<br>Adequate funding	High speed of service set-up	Nurtures commitment to partnership<br>(Faith in collaboration)	I2: 13
1	The willingness of partner organisations to participate in the planning, implementation and improvement of service	Rapid establishment of GTTPS	(Feeling that ability to work together is the key success of service)	I3: 4
1		Quick running of service	Feeling that ability to work together is critical to success	I6: 11-12
1	Commitment to a common aim, willingness to invest time, streamlining bureaucratic procedures, and working outcome-focused	Successful set-up of service, very quickly with very little knowledge	(Feeling that ability to work together is the key to the success of service)	I8: 10

* Partnership stages:
1 Formation: Identification of partners, the definition of a common aim, partnership structure
2 Development: Set up of partnership structures, building relationships
3 Solidification: Solidification of partnership structures and relations
