# Supplementary material for: Trust-building in temporary public health partnerships: a qualitative study of the partnership formation process of a Covid-19 test, trace and protect service
Source: BMC Health Serv Res. 2024 Apr 13;24:467. doi: 10.1186/s12913-024-10930-3 (PMC11015697; doi:10.1186/s12913-024-10930-3)
Supplement: Supplementary file 2 — Additional file 2. Summary of trust-building processes emanating from content analysis. Summary of drivers for initiating and sustaining trusting partnership relations resulting from an exploration of the interplay between different variables extracted from data analysis. [file 12913_2024_10930_MOESM2_ESM.pdf]

**Additional Table 2** Summary of trust-building processes emanating from content analysis

| Stage* | Theme | Cause | Effect | Source |
|---|---|---|---|---|
| **Initiating the trust-building loop** | | | | |
| **1. Forming expectations** | | | | |
| ***1.1 Previous experiences of collaboration*** | | | | |
| 2 | Structured set-up of service and started operating quickly | Previous positive experiences in working together | Increased faith in collaboration | I10, I13, I14 |
| 1,2 | Familiarity, trust and acceptance between partners | Existing good relationships between partners, Personal networks | Faith in cooperative behaviour | I3, I9 |
| ***1.2 Need for collaboration*** | | | | |
| 1 | Need to pool information, knowledge and expertise | Threat, urgency and unpredictability of the pandemic situation | Willing to collaborate, Commitment to partnership | I1, I3, I11 |
| 1 | Need to pool personnel and financial resources | Need for resources | Willing to collaborate, Commitment to partnership | I1, I9 |
| 1 | Facing the same difficulties | The threat, urgency, and unpredictability of the pandemic situation | Unifying effect Willing to support each other | I9, I12 |
| ***1.3 Commitment to a common aim*** | | | | |
| 2 | Workforce highly committed to a common aim. | Recruitment and personnel development strategies, Leadership behaviour (Role modelling) | Easy relationship building, Big team feeling | I1, I4, I6, I7 I13, NHS 360 leadership development |
| 2 | Leaders highly committed to common aims and partnership | Leadership Behaviour | Trust in leaders | I9, NHS 360 leadership development |
| 3 | Less commitment to partnership | Different priorities, less urgency of purpose, funding concerns | Less willingness to collaborate | I2 |
| **2. Managing risk** | | | | |
| ***2.1 Collaborative set-up of the service*** | | | | |
| 1 | The collaborative, supportive nature of service set-up | Commitment to common aim and partnership | Faith in collaboration Big team feeling | I2, I3, I7, I9, I10, I12 |

| Stage* | Theme | Cause | Effect | Source |
|---|---|---|---|---|
| **Sustaining the trust-building loop** | | | | |
| **1. Managing power imbalances** | | | | |
| ***1.1 Governance structure*** | | | | |
| 2, 3 | Shared decision-making forum (funding and resources) | Equal representation of all partner organisations in the Leadership group | Commitment to partnership and common aim, Trust in governance structures | I1, I2, I3, I5, I9, I12, governance framework |
| 2 | Coordination Unit situated at the local authority | Avoid unequal power distribution among partner organisations | Acceptance and trust in governance structures | I1, I2 |
| 1, 2, 3 | Local ownership of partner organisations | Decentralised service structures | Flexibility in decision-making<br>Acceptance and trust in governance structures | I9, I12 |
| ***1.2 Shared decision-making*** | | | | |
| 2,3 | Supportive, approachable staff | Network communication | Support network, Close working bonds | I4, I5, I7, I9, I13, S79 |
| 1 | Collective decision-making | Emergency | Shared decision-making<br>Equal power distribution | I2, I3 |
| 1, 2, 3 | Solution-oriented decision-making without hierarchical thinking | Leadership behaviour and attitudes | Equal power distribution | I1, I2, I4, I6, I10 |
| *2, 3* | Acknowledgement of partner views and contributions | Leadership behaviour and attitudes | Trusting relationships<br>Local ownership | I7, I10, I12 |
| ***1.3 Dealing with power imbalances*** | | | | |
| 2,3 | Tensions aroused from central control | Governance structure based on support rather than control | Acceptance and trust in governance structures | I1, I6, I7, I9, governance framework |
| ***1.4 Dealing with tension and failure*** | | | | |
| 2, 3 | Acknowledgement of partner views and contributions | Leadership behaviour and attitudes | Trusting relationships<br>Local ownership | I7, I10, I12 |
| *1, 2, 3* | Open failure culture | Acceptance of failure<br>Limited bureaucracy | Feeling safe and trusted | I2, I3, I4, I9, I10 |

| Stage* | Theme | Cause | Effect | Source |
|---|---|---|---|---|

| | | | | |
|---|---|---|---|---|
| **2. Nurturing the collaborative relationships** | | | | |
| ***2.1 Network communication*** | | | | |
| *2* | Frequent communication across partner organisations | High frequency of formal and informal meetings | Mutual understanding, High-quality relationships | I2, I3, I7, I8, I9, I13 |
| *2, 3* | Smooth communication across partner organisations | Flat organisational hierarchy | Networking, High-quality relationships | I4, I5 |
| *2* | Understanding the roles and tasks of partner organisations | Changing positions within the partnership | Mutual understanding, High-quality relationships | I10, I13 |
| ***2.2 Approachable, supportive workforce*** | | | | |
| *2, 3* | Supportive, approachable staff | Flat organisational structure<br>Less hierarchical thinking | Support network | I2, I4, I5, I6, I13, S 79 |
| *2, 3* | Supportive, approachable staff | Network communication | Support network, Close working bonds | I4, I5, I7, I9, I13, S79 |
| *2, 3* | Supportive, approachable staff | Personalities of leaders and staff | Support network, Close working bonds | I6, I7, I12, I14 |
| *2, 3* | Supportive, approachable staff | Commitment to a common aim | Close working bonds, Mutual recognition | I6, I7, I9, I13 |
| ***2.3 Situational awareness*** | | | | |
| 2, 3 | Creating situational awareness | High frequency of meetings<br>Two-way communication in meetings | Feeling connected, supported and involved<br>Feeling safe and trusted | I1, I3, I5, I6, I13 |
| ***2.4 Recognition and respect*** | | | | |
| 2, 3 | Recognition and respect among a highly diverse workforce | Leadership behaviour (role modelling) | Strengthening relations based on mutual acceptance<br>Big team feeling | I2, I10, I11 |
| 2, 3 | Recognition and respect among a highly diverse workforce | Network communication<br>Mutual support,<br>Sharing knowledge and expertise<br>Commitment to a common aim | Strengthening relations based on mutual acceptance<br>Big team feeling | I2, I3, I4, I6, I7, I11, I13, S89 |
| Stage* | Theme | Cause | Effect | Source |

***2.5 Gaining underpinnings for more ambitious collaboration***

| | | | | |
|---|---|---|---|---|
| 2, 3 | Service performance | Commitment to common aim and partnership | Faith in collaboration Big team feeling | I1, I2, I6, I10 |
| 3 | Knowledge gains | Collaboration within and across partner organisations | Organisational learning, Individual learning experience, Big team feeling | I4, I5, I6, I7, I13, S31 |

*Partnership stages:
1 Formation: Identification of partners, the definition of a common aim, partnership structure
2 Development: Set up of partnership structures, building relationships
3 Solidification: Solidification of partnership structures and relations
